# Indirect genetic effects and inbreeding: consequences of BLUP selection for socially affected traits on rate of inbreeding

**DOI:** 10.1186/1297-9686-46-39

**Published:** 2014-06-24

**Authors:** Hooi Ling Khaw, Raul W Ponzoni, Piter Bijma

**Affiliations:** 1 Animal Breeding and Genomics Centre, Wageningen University, De Elst 1, 6708WD Wageningen, The Netherlands; 2 WorldFish, Jalan Batu Maung, 11960 Bayan Lepas, Penang, Malaysia; 3 Departamento de Produccion Animal, Facultad de Agronomia, 12900 Montecideo, Uruguay

## Abstract

**Background:**

Social interactions often occur among living organisms, including aquatic animals. There is empirical evidence showing that social interactions may genetically affect phenotypes of individuals and their group mates. In this context, the heritable effect of an individual on the phenotype of another individual is known as an Indirect Genetic Effect (IGE). Selection for socially affected traits may increase response to artificial selection, but also affect rate of inbreeding.

**Methods:**

A simulation study was conducted to examine the effect of Best Linear Unbiased Prediction (BLUP) selection for socially affected traits on the rate of inbreeding. A base scenario without IGE and three alternative scenarios with different magnitudes of IGE were simulated. In each generation, 25 sires and 50 dams were mated, producing eight progeny per dam. The population was selected for 20 generations using BLUP. Individuals were randomly assigned to groups of eight members in each generation, with two families per group, each contributing four individuals. “Heritabilities” (for both direct and indirect genetic effects) were equal to 0.1, 0.3 or 0.5, and direct–indirect genetic correlations were −0.8, −0.4, 0, 0.4, or 0.8. The rate of inbreeding was calculated from generation 10 to 20.

**Results:**

For the base scenario, the rates of inbreeding were 4.09, 2.80 and 1.95% for “heritabilities” of 0.1, 0.3 and 0.5, respectively. Overall, rates of inbreeding for the three scenarios with IGE ranged from 2.21 to 5.76% and were greater than for the base scenarios. The results show that social interaction within groups of two families increases the resemblance between estimated breeding values of relatives, which, in turn, increases the rate of inbreeding.

**Conclusion:**

BLUP selection for socially affected traits increased the rate of inbreeding. To maintain inbreeding at an acceptable rate, a selection algorithm that restricts the increase in mean kinship, such as optimum contribution selection, is required.

## Background

Aquaculture produces fish at an affordable price that are a valuable source of animal proteins, especially in developing countries [1]. Selective breeding plays an important role in aquaculture and provides high quality seed with better growth rate and survival [2]. Genetically Improved Farmed Tilapia (GIFT) in tropical countries [3,4], and Atlantic salmon in temperate and cold countries [2] are good examples that illustrate the benefits of selective breeding. However, there is evidence that fish with high growth rate may be more aggressive and competitive [5,6]. Competition is a type of social interaction that is very common in aquaculture environments. It reduces productivity and represents a threat to animal welfare [7,8]. Thus, fish breeders may need to improve productivity and welfare by taking social interactions into account in their breeding programs.

In the absence of social interactions among individuals, the phenotypic value (*P*_*i*_) of an individual, say *i*, can be modeled as the sum of its additive genetic or breeding value (*A*_*i*_), and a non-genetic component, usually referred to as environment (*E*_*i*_) [9],

(1) $$P_{i}=A_{i}+E_{i}.$$

Using this model, breeders have achieved substantial genetic improvement. However, in some cases, especially for traits related to behavior, populations have not responded as expected, in spite of the presence of heritable variation. For example, selection for survival increased mortality in laying hens [10]. One of the reasons for these unexpected responses may be the presence of indirect genetic effects (IGE). An IGE is a heritable effect of an individual on the trait value of another individual [10-13]. For example, in fish, when an individual carries genes that cause it to monopolize the feeder, the growth rate of its group mates will be reduced. Another well-known example is mortality due to cannibalism in laying hens, where the survival of an individual depends on the genes for pecking behaviour in its cage mates [10].

For such traits, the model in Equation (1) needs to be expanded with IGE [11,13,14],

(2) $$P_{i}=A_{D,i}+E_{D,i}+\sum_{j\neq i}^{n-1}A_{S,j}+\sum_{j\neq i}^{n-1}E_{S,j},$$

where, *P*_*i*_ is the phenotype of focal individual *i*; *A*_*D*,*i*_ and *E*_*D*,*i*_ are the direct breeding value and direct non-genetic effect of individual *i*, respectively; *A*_*S*,*j*_ and *E*_*S*,*j*_ are the indirect breeding value and indirect non-genetic effect originating from its group mate *j*, respectively; and *n* is the group size. The summations are taken over the *n*-1 group mates of an individual, thus excluding *i*. This model applies to all *n* group members. From the perspective of the recipient, each individual’s phenotype is the consequence of a direct effect of itself, and the sum of the indirect effects of its *n*-1 social partners. From the perspective of the acting individual, each individual expresses its direct genetic effect once in its own phenotype, and its IGE *n*-1 times, once in each of its *n*-1 group mates. Thus, in addition to the classical (direct) breeding value (Equation 1), each individual affects its *n*-1 group mates, and is also affected by its *n*-1 group mates. Several studies have shown the existence of IGE, in quail [15], poultry [16-18], pigs [19,20], cattle [21], and fish [22].

Theoretical and empirical studies show that response to selection for socially affected traits can be increased by applying a selection strategy that accounts for both direct and indirect genetic effects, such as kin or group selection [10,13,14,17,23,24]. Muir [10], for example, conducted a selection experiment for egg production in cannibalistic laying hens using group selection, and managed to increase egg production from 91 to 237 eggs per hen, largely as result of improved survival. There have been several other selection experiments on socially affected traits, for example, in quail [24] and flour beetles [25,26]. To our knowledge, there have been no similar studies in aquaculture species.

Theoretical and experimental work on socially affected traits shows that response to selection can be increased by using structured populations with groups composed of related individuals. In such populations, response to selection is maximized by selecting on the Best Linear Unbiased Prediction (BLUP) of breeding values [13]. However, such selection schemes may lead to high rates of inbreeding, because they increase the probability of co-selection of relatives [27,28]. High rates of inbreeding cause a reduction of genetic variance and threaten the long-term sustainability of breeding programs [29-32].

In this study, we examined the effect of BLUP selection for a socially affected trait on the rate of inbreeding for a fish breeding program using stochastic simulation. However, the methods and results are also applicable to other species with similar breeding designs.

## Methods

### Population structure

Data were simulated using the R-language [33]. The simulated population was a closed nucleus with discrete generations in which base population animals were assumed unrelated. Bivariate normal distributions were used to simulate both the genetic and the non-genetic direct and indirect effects of base animals. Subsequently, in each generation, 25 sires and 50 dams were selected and randomly mated. Each male was mated to two females in a nested design, which is a common mating structure in aquaculture breeding programs. Each dam produced eight progeny and the sex of the progeny was randomly assigned with equal probability.

Direct and indirect breeding values of an offspring were simulated as the average breeding value of its parents plus a Mendelian sampling deviation, sampled from a bivariate normal distribution. Individuals in each generation were assigned to groups of eight members, with a group consisting of two full-sib families and each family contributing four progeny. Subsequently, phenotypes of individuals were constructed according to Equation 1 and breeding values were estimated (see below). We chose a design with two families per group because this scheme is optimal to estimate the indirect genetic variance [34] and yields greater response to selection than schemes with groups composed at random with respect to family [14,17]. Schemes with a single family per group would yield an even greater response, but would not allow the estimation of the direct and indirect genetic variances [16,21,35]. Hence, having two families per group appears to be an attractive compromise for response to selection and variance component estimation.

The top 25 male candidates and top 50 female candidates were selected as parents of the next generation based on the BLUP estimate of their total breeding value ($T\hat{B}V$),

(3) $$T\hat{B}V={\hat{A}}_{D}+\left(n-1\right){\hat{A}}_{S},$$

where ${\hat{A}}_{D}$ and ${\hat{A}}_{S}$ are the estimated direct and indirect breeding values, respectively, and *n* is group size [14]. For each scenario, 20 generations of selection were simulated.

The BLUP estimated breeding values (EBV) were obtained from the following model [13,14], using the R-version of ASReml [36]:

(4) $$y=\mu +Z_{D}a_{D}+Z_{S}a_{S}+\mathrm{Vg}+e,$$

where **y** is the vector of phenotypic observations; *μ* is the overall mean, which was the only fixed effect included; **a**_*D*_ and **a**_*S*_ are the vectors of direct and indirect random genetic effects, respectively; **g** is the vector of random group effects, and **e** is the vector of random residuals. The random group effects in **g** occur as a result of the non-genetic indirect effects (*E*_*S*_ in Equation 2), which create a covariance among group members that can be fitted as a group effect. The magnitude of this covariance equals $\sigma_{g}^{2}=2\sigma_{E_{\mathrm{DS}}}+\left(n-2\right)\sigma_{E_{S}}^{2}$, where $\sigma_{E_{\mathrm{DS}}}$ is the direct–indirect non-genetic covariance and $\sigma_{E_{S}}^{2}$ is the non-genetic indirect variance [19]. Thus, $\sigma_{g}^{2}$ is determined by the phenotypic variances, heritabilities and non-genetic correlations that were used as input values for the simulations (see Table 1 below). The **Z**_*D*_, **Z**_*S*_, and **V** are the known design matrices that assign observations to the levels of the direct genetic effects of the animals themselves, to the IGE of their group mates, and to the random group effects, respectively. The **Z**_S_-matrix has a “1” in the column for each group mate of the individual producing the record. Hence, since the group size was equal to 8 in our data, each row of **Z**_S_ contains seven 1 s, each linking the IGE of one group mate to the record of the individual. The covariance structure of the random effects was:

$$\mathrm{var}\left[\begin{array}{c} a_{D}\\ a_{S}\\ g\\ e \end{array}\right]=\left[\begin{array}{cccc} A\sigma_{A_{D}}^{2} & A\sigma_{A_{\mathrm{DS}}} & 0 & 0\\ A\sigma_{A_{\mathrm{DS}}} & A\sigma_{A_{S}}^{2} & 0 & 0\\ 0 & 0 & I\sigma_{g}^{2} & 0\\ 0 & 0 & 0 & I\sigma_{e}^{2} \end{array}\right].$$

**Table 1:** Assumed parameters used in simulation by scenario

**Parameters**	**Scenarios**			
	**Base**	**1**	**2**	**3**
^a^Magnitude of indirect effect, $\left(n-1\right)\sigma_{P_{S}}^{2}$	0	0.25	1.0	4.0
Correlations between direct and indirect effects, $r_{A_{\mathrm{DS}}}=r_{E_{\mathrm{DS}}}$	0	0, −0.8, −0.4, 0.4, 0.8	0, −0.8, −0.4, 0.4, 0.8	0, −0.8, −0.4, 0.4, 0.8
Direct phenotypic variance, $\sigma_{P_{D}}^{2}$	1	1	1	1
^b^Heritabilities, $h_{D}^{2}=h_{S}^{2}$	0.1, 0.3, 0.5	0.1, 0.3, 0.5	0.1, 0.3, 0.5	0.1, 0.3, 0.5

^a^Since an individual interacts with *n*-1 group mates, the term $\left(n-1\right)\sigma_{P_{S}}^{2}$ reflects the contribution of indirect effects (both genetic and non-genetic) to phenotypic variance; ^b^Heritabilities are the ratio of additive genetic variance to the corresponding “phenotypic” variance; for direct effects $h_{D}^{2}=\sigma_{A_{D}}^{2}/\sigma_{P_{D}}^{2}$, and for indirect effects $h_{S}^{2}=\sigma_{A_{S}}^{2}/\sigma_{P_{S}}^{2}$.

In the estimation of breeding values, the true (*i.e.*, simulated) values of the genetic parameters were used. Genetic parameters were not estimated from the simulated data.

### Rate of inbreeding

The inbreeding coefficients of individuals were calculated from the pedigree by using the R-package “pedigree” [37]. For each replicate, the rate of inbreeding (*ΔF*) was then calculated using the mean inbreeding coefficients of generations 10 and 20:

(5) $$\mathrm{\Delta F}=1-\sqrt[{10}]{\frac{1-{\bar{F}}_{20}}{1-{\bar{F}}_{10}}}.$$

Rates of inbreeding were averaged over 100 replicates and the standard error was calculated. The first 10 generations were not used in the calculation of the rate of inbreeding, to allow the population to reach equilibrium with respect to the Bulmer effect and the buildup of pedigree information [38-40]. The Bulmer effect reduces the between-family variance, which reduces the correlation between EBV of relatives. This, in turn, reduces the probability of co-selection of relatives, which reduces the rate of inbreeding. Thus, the Bulmer-effect affects the rate of inbreeding [41].

### Simulated scenarios

A base scenario and three alternatives were simulated (Table 1). In all schemes, the direct phenotypic variance was set to 1, $\sigma_{P_{D}}^{2}=\sigma_{A_{D}}^{2}+\sigma_{E_{D}}^{2}=1$. The base scenario was a reference scenario without indirect effects (genetic and non-genetic), where trait values were generated according to Equation 1. The alternative scenarios considered different magnitudes of indirect effects: mild (scenario 1), intermediate (scenario 2) or strong (scenario 3). The magnitude of indirect effects was measured by their contribution to phenotypic variance in a population in which interacting individuals are unrelated, which is given by $\left(n-1\right)\sigma_{P_{S}}^{2}$ and was equal to 0.25, 1, or 4 ($\sigma_{P_{S}}^{2}=\sigma_{A_{S}}^{2}+\sigma_{E_{S}}^{2}$). Thus, compared to direct effects, the contribution of indirect effects was equal to one quarter to four-fold the direct phenotypic variance ($\sigma_{P_{D}}^{2}$) to phenotypic variance. For all scenarios, “heritabilities” of direct and indirect genetic effects were equal to 0.1, 0.3 or 0.5 ($h_{D}^{2}=\sigma_{A_{D}}^{2}/\sigma_{P_{D}}^{2}$ and $h_{S}^{2}=\sigma_{A_{S}}^{2}/\sigma_{P_{S}}^{2}$). Genetic and non-genetic correlations between direct and indirect effects were varied as follows: $r_{A_{\mathrm{DS}}}$ = $r_{E_{\mathrm{DS}}}$ = −0.8, −0.4, 0, 0.4 or 0.8.

## Results

Across the four scenarios, rates of inbreeding ranged from 2.21 to 5.76%. The standard errors of the rates of inbreeding (average over 100 replicates) were small and ranged from 0.0004 to 0.0014, which indicates that the results were accurate.

For presentation purposes, the results were grouped according to the correlation between direct and indirect effects:

a. Neutral, the direct–indirect correlations were equal to zero ($r_{A_{\mathrm{DS}}}=r_{E_{\mathrm{DS}}}$ = 0);

b. Competition, the correlations were negative ($r_{A_{\mathrm{DS}}}=r_{E_{\mathrm{DS}}}$ = −0.4 and −0.8);

c. Cooperation, the correlations were positive ($r_{A_{\mathrm{DS}}}=r_{E_{\mathrm{DS}}}$ = 0.4 and 0.8).

**Figure 1:**
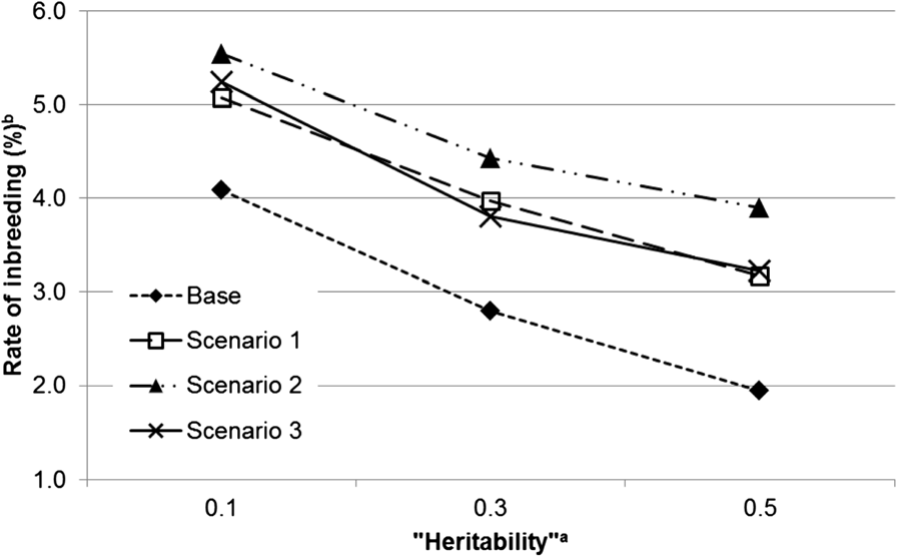
**Rates of inbreeding (%) for the four scenarios across heritabilities**^**a **^**when correlations between direct and indirect genetic effects are equal to 0. **^a^Heritabilities are the ratio of additive genetic variance to the corresponding “phenotypic” variance; for direct effects $h_{D}^{2}=\sigma_{A_{D}}^{2}/\sigma_{P_{D}}^{2}$, and for indirect effects $h_{S}^{2}=\sigma_{A_{S}}^{2}/\sigma_{P_{S}}^{2}$; ^b^the SE of rate of inbreeding ranged between 0.00039 and 0.00125.

**Figure 2:**
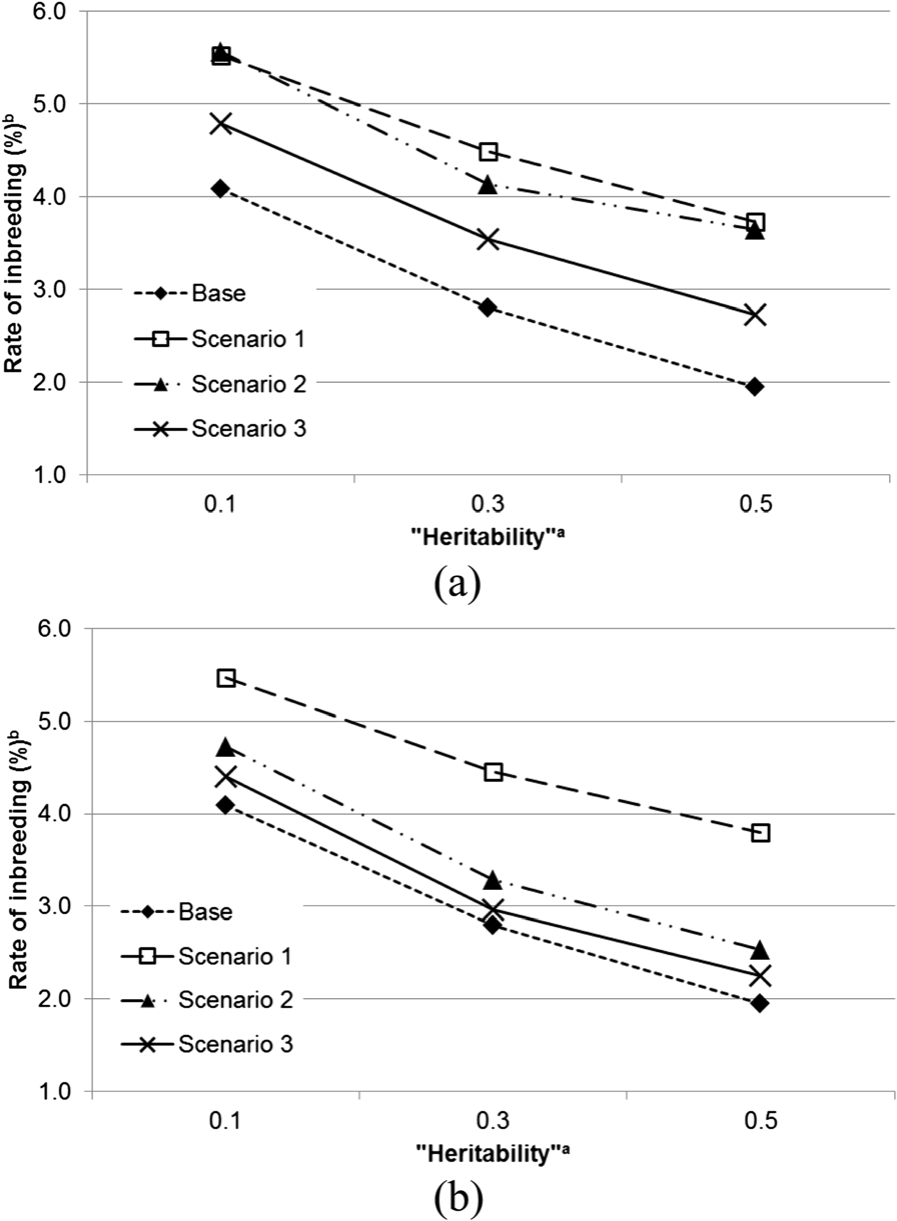
**Rates of inbreeding (%) for the scenarios across heritabilities**^**a **^**when correlations between direct and indirect genetic effects are equal to −0.4 (a) and −0.8 (b), except for the base scenario. **^a^Heritabilities are the ratio of additive genetic variance to the corresponding “phenotypic” variance; for direct effects $h_{D}^{2}=\sigma_{A_{D}}^{2}/\sigma_{P_{D}}^{2}$, and for indirect effects $h_{S}^{2}=\sigma_{A_{S}}^{2}/\sigma_{P_{S}}^{2}$; ^b^the SE of rate of inbreeding ranged between 0.00046 and 0.00139.

**Figure 3:**
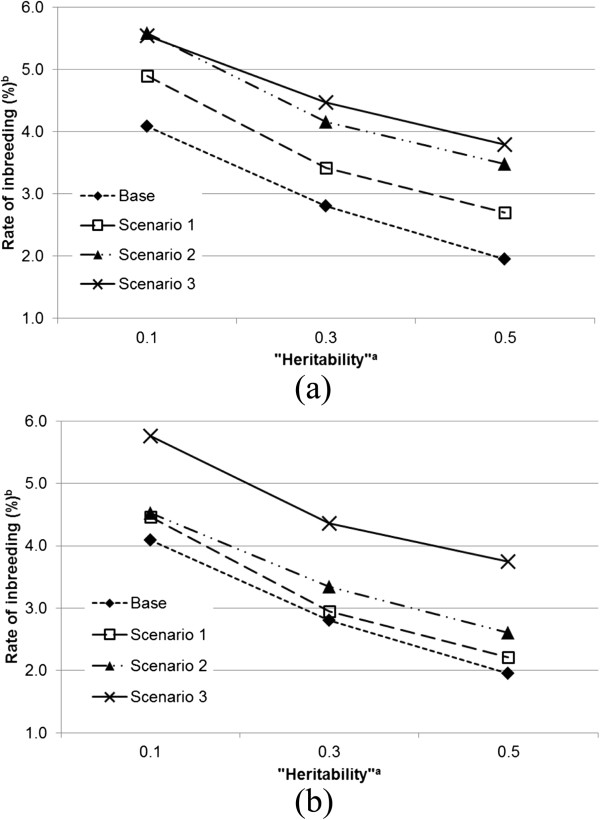
**Rates of inbreeding (%) for the scenarios across heritabilities**^**a **^**when correlations between direct and indirect genetic effects are equal to 0.4 (a) and 0.8 (b), except for the base scenario. **^a^Heritabilities are the ratio of additive genetic variance to the corresponding “phenotypic” variance; for direct effects $h_{D}^{2}=\sigma_{A_{D}}^{2}/\sigma_{P_{D}}^{2}$, and for indirect effects $h_{S}^{2}=\sigma_{A_{S}}^{2}/\sigma_{P_{S}}^{2}$; ^b^the SE of rate of inbreeding ranged between 0.00038 and 0.00129.

Under the neutral situation, the direct effect of an individual on its own trait value is independent of its indirect effect on the trait values of its group mates. Figure 1 shows the results for this situation. Rates of inbreeding were always greater for scenarios with IGE than with the base scenario. The range for rates of inbreeding obtained from scenarios 1, 2 and 3 was 3.17 to 5.54%, and from 1.95 to 4.09% for the base scenario. The rates of inbreeding within each scenario were greatest with a low “heritability” (*i.e.*, lower values of $h_{D}^{2}=\sigma_{A_{D}}^{2}/\sigma_{P_{D}}^{2}$ and $h_{S}^{2}=\sigma_{A_{S}}^{2}/\sigma_{P_{S}}^{2}$).In a competitive situation, an individual with positive effects on its own trait value will on average have negative effects on the trait values of its group mates (Figure 2a and b). In this situation, the rate of inbreeding was lowest with the base scenario and highest with scenario 1. Rates of inbreeding were almost identical for both direct–indirect correlations with scenario 1. The rates of inbreeding for scenario 2 were between those for scenarios 1 and 3. However, note that in scenario 2, a change in the direct–indirect correlation had a greater effect on rates of inbreeding than in the other scenarios. The lowest rates of inbreeding were obtained from scenario 3 and rates of inbreeding decreased when the correlation changed from −0.4 to −0.8.In the cooperative situation, an individual with positive effects on its own trait value also has positive effects on the trait values of its group mates (Figure 3a and b). Apart from the base scenario, which again produced the lowest inbreeding rate, ranking of scenarios with respect to rate of inbreeding was precisely opposite for this situation to that obtained from the competitive situation. The highest rate of inbreeding was obtained from scenario 3 and the lowest from scenario 1. Scenario 3 also showed the most stable rates of inbreeding across different direct–indirect correlations. As was the case in the competitive situation, scenario 2 was the most sensitive to a change in the value of the direct–indirect correlation.

## Discussion

### Overall findings

Our results indicate that BLUP selection on socially affected traits results in greater rates of inbreeding than BLUP selection solely for direct genetic effect, regardless of the genetic correlations between direct and indirect genetic effects. Furthermore, the pattern of the rates of inbreeding for different “heritabilities” was in agreement with BLUP selection theory, with lower heritability yielding higher rates of inbreeding [27,28].

### Rate of inbreeding and BLUP selection

For decades, inbreeding has been identified as an important issue in animal breeding [9,32,42]. Artificial selection is known to increase the rate of inbreeding because individuals from the best performing families are selected and contribute more to the gene pool compared to those from lower performing families [32,42-44], which is confirmed by our results. Without selection, the expected rate of inbreeding for the simulated population is about 0.75% per generation (using $\mathrm{\Delta F}=\frac{1}{8N_{m}}+\frac{1}{8N_{f}}$, with *N*_*m*_ = 25 and *N*_*f*_ = 50). In our study, the rates of inbreeding (2.21 to 5.76% with IGE, and 1.95 to 4.09% without IGE) were considerably higher. Furthermore, the highest rates of inbreeding were obtained with low heritabilities. This is as expected with BLUP selection, since information from relatives receives higher weight with low heritabilities, which increases the probability of co-selection of relatives, thus increasing the rate of inbreeding [27,28].

To investigate whether lower heritabilities can explain the higher rates of inbreeding observed in scenarios with IGE, we calculated the classical (*i.e.* direct) heritability, $\sigma_{A_{D}}^{2}/\sigma_{P}^{2}$, for the four scenarios under the neutral situation, for values of $h_{D}^{2}=\sigma_{A_{D}}^{2}/\sigma_{P_{D}}^{2}$ = $h_{S}^{2}=\sigma_{A_{S}}^{2}/\sigma_{P_{S}}^{2}$ = 0.3. Phenotypic variance for groups composed of two families was calculated as σP2=σAD2+n−1σAS2+n−2rσADS+212n−112n−1rσAS2+σED2+n−1σES2, where *r* = 0.5 is the relationship between members of the same family. The three scenarios with IGE had lower classical heritabilities than the base scenario (classical heritability of 0.3). For example, with scenario 2, classical heritability was 0.3/2.39 = 0.13 (see Table 1 for the parameters used) and with scenarios 1 and 3, it was equal to 0.22 and 0.05, respectively. When comparing these classical heritabilities to the observed rates of inbreeding, the pattern was different from that observed with classical BLUP selection for direct effects only. The heritability for scenario 2 was in between those with scenarios 1 and 3, yet scenario 2 had the highest rate of inbreeding. Thus, apart from a potential effect working via classical heritability, IGE also affects the rate of inbreeding in other ways.

However, based on these results, we cannot determine whether the increase in rate of inbreeding in scenarios 1 to 3 compared to the base scenario was caused by IGE or by a reduction in classical heritability due to extra variance. Therefore, we simulated an additional scheme with classical heritability fixed at 0.3 by increasing the direct genetic variance ($\sigma_{A_{D}}^{2}$) for scenarios 1 and 2, while the genetic and non-genetic indirect effects remained unchanged. A comparison of the rate of inbreeding of this scheme to that of the base scenario (also for $h_{D}^{2}=0.3$) reveals the impact of IGE on the rate of inbreeding at a fixed classical heritability. For this additional scheme, the rates of inbreeding for scenarios 1 and 2 were equal to 3.62 and 3.86%, respectively, which was about 1% higher than in the base scenario (2.80%). Based on this result, we can confidently conclude that the indirect effect was a causal factor that contributed to the increase of the rate of inbreeding. Scenario 3 was not included as an additional scheme, because $h_{D}^{2}$ would have to be greater than 1 to achieve a classical heritability of 0.3 for that scenario.

### Competition versus cooperation

**Figure 4:**
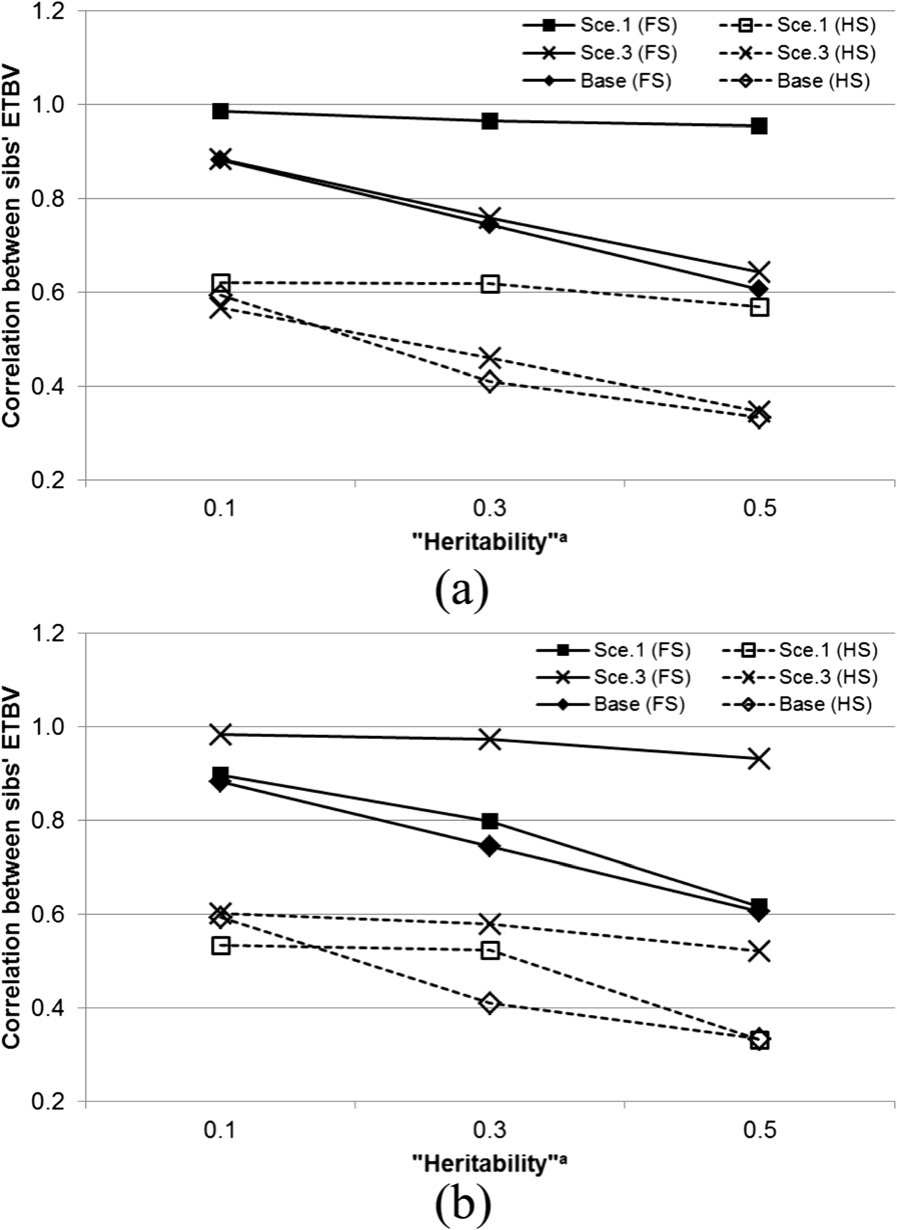
**Correlations between estimated total breeding values (ETBV) for full-sibs (FS) and for half-sibs (HS) for a correlation between direct and indirect genetic effects of −0.8 (a) or +0.8 (b). **^a^Heritabilities are the ratio of additive genetic variance to the corresponding “phenotypic” variance; for direct effects $h_{D}^{2}=\sigma_{A_{D}}^{2}/\sigma_{P_{D}}^{2}$, and for indirect effects $h_{S}^{2}=\sigma_{A_{S}}^{2}/\sigma_{P_{S}}^{2}$.

Comparing the competitive (Figure 2) and cooperative (Figure 3) situations, we observed a re-ranking of scenarios 1 and 3. To understand the mechanisms behind these results, we calculated the correlations between the estimated total breeding values (ETBV) for full-sibs and half-sibs, for direct–indirect correlations of −0.8 (Figure 4a) and +0.8 (Figure 4b). The results show that the re-ranking of scenarios observed for the rate of inbreeding was mirrored in the correlation between ETBV of sibs. The highest correlation between ETBV of sibs was obtained from scenario 1 for a direct–indirect correlation of −0.8 and from scenario 3 for a direct–indirect correlation of +0.8. These results suggest that the correlation between ETBV of sibs is the main cause for the differences in rates of inbreeding. A higher correlation between ETBV of sibs increases the probability of co-selection of sibs, which, in turn, increases the rate of inbreeding because it increases the variance in long-term contributions of ancestors [44]. Nevertheless, the correlation between ETBV of sibs did not fully explain the observed pattern of rates of inbreeding. In Figure 4a, for example, the correlation between ETVB of sibs was nearly independent of “heritability” for scenario 1, but this trend was not reflected in the rate of inbreeding (Figure 1). The observed pattern for the correlation between ETBV of sibs for the base scenario was similar to its pattern for rate of inbreeding.

In traditional selection on BLUP EBV, rates of inbreeding and correlations between EBV of sibs are higher at lower heritability. Thus, it was interesting to investigate whether the same mechanism explains the re-ranking of scenarios 1 and 3 observed here. Therefore, we analyzed the relationship of the ratio of total heritable variance over phenotypic variance, $T^{2}=\frac{\sigma_{\mathrm{TBV}}^{2}}{\sigma_{P}^{2}}$, with the rate of inbreeding. For the competitive situation, a lower *T*^2^ indeed corresponded to a higher rate of inbreeding. For example, with $r_{g}=-0.8\left(h_{D}^{2}=h_{S}^{2}=0.1\right)$, values obtained from scenarios 1 and 3 were $T_{1}^{2}$ = 0.05 and $T_{3}^{2}$ = 0.39, and scenario 1 yielded a greater rate of inbreeding than scenario 3 (Figure 2b). However, for the cooperative situation, the rate of inbreeding increased when *T*^2^ increased. For example, with *r*_*g*_ = 0.8 ($h_{D}^{2}=h_{S}^{2}=0.1$), values obtained from scenarios 1 and 3 were $T_{1}^{2}$ = 0.37 and $T_{3}^{2}$ = 0.66, and the rates of inbreeding for scenario 3 were greater than for scenario 1 (Figure 3b).

The above results on the relationship between inbreeding with classical heritability and *T*^2^ show that patterns observed with selection on classical BLUP-EBV cannot simply be extended to schemes that aim at improving socially affected traits. One reason is that the correlation between EBV of sibs is no longer a simple function of heritability, but depends also on the direct–indirect genetic correlation and on relatedness between group mates. In principle, the theory of long-term genetic contributions [41,44] can be used to predict the rate of inbreeding for socially affected traits using a deterministic approach, similar to its application to selection on traditional animal model BLUP EBV [28]. However, this requires a pseudo-BLUP selection index [45] for socially affected traits. Although this is relatively straight-forward in principle, the full single-trait pseudo-BLUP selection index for a socially affected trait with sib information has 24 distinct information sources (results not shown). Hence, deterministic prediction of the rate of inbreeding with social effects is feasible but cumbersome, and one may prefer to use stochastic simulations instead.

### Relevance of the results to other situations

This study focused on breeding schemes in aquaculture. However, the results are also relevant for other species in which selection is based on sib information. In our simulations, we covered a wide-range of values with respect to the magnitude of indirect effects, heritabilities and direct–indirect genetic correlations. For all scenarios, presence of IGE increased the rate of inbreeding. Moreover, our group size of eight individuals is similar to group sizes in pigs and laying hens bred in cage systems. Furthermore, the design with two families per group is optimal for maximizing response to selection while maintaining the opportunity to estimate genetic parameters [34], and is thus relevant for any breeding scheme for traits affected by IGE. However, the strategy of mating one male to two females is typical for breeding programs applied on some aquaculture species but it is uncommon in livestock. We do not expect that the mating ratio will substantially change the impact of IGE on the rate of inbreeding in sib selection schemes. Hence, we postulate that the main result of this study, which is the presence of IGE increases rates of inbreeding, can be extended to sib selection schemes in other species.

### Solutions and future direction to manage rates of inbreeding

Because rates of inbreeding are greater with IGE, breeding programs that aim at improving socially affected traits require greater effort to contain inbreeding, which means that more genetic gain has to be sacrificed. Optimum contribution selection [46] is the best method to restrict the rate of inbreeding while maximizing genetic gain, and implementation to traits affected by IGE is straightforward. Compared to current breeding schemes in aquaculture, which often rely on sib information, the use of genomic selection will decrease the correlation between EBV of sibs. Hence, we anticipate that the cost of restricting inbreeding will be reduced with genomic selection, and, for that reason, aquaculture breeding programs for socially affected traits would also benefit from genomic selection.

The feasibility of BLUP and optimum contribution selection, however, depends on the availability of pedigree or genomic information [46]. Aquaculture breeding programs in developing countries are generally faced with difficulties to maintain a fully pedigreed structure because it is too costly to individually identify the fish. When pedigree or genomic information is not available, breeders have to rely on mass selection. IGE will not affect the rate of inbreeding with mass selection when groups are composed at random with respect to relatedness because IGE do not affect the ranking of individuals in this case [14]. However, genetic improvement of traits affected by IGE using mass selection is efficient only when the population is structured into groups consisting of families [14,23]. Such schemes will also increase the resemblance between phenotypes of relatives and thus lead to increased rates of inbreeding when mass selection is simply by truncation based on the observed phenotype. Hence, in those cases, breeders will have to restrict the contribution of individual families to the next generation, which is less efficient and will yield further reduction in genetic gain compared to full optimal contribution selection.

## Conclusions

Our study shows that BLUP selection for socially affected traits increases the rate of inbreeding compared to traditional BLUP selection. This is at least partly due to the greater resemblance between EBV of relatives when animals are kept in groups consisting of two families. When accounting for IGE in a selection program, measures have to be taken to limit the rate of increase in mean kinship. Such measures may include optimum contribution selection, or limiting the number of candidates selected from each family.

## Competing interests

The authors declare that they have no competing interest.

## Authors’ contributions

HLK carried out the simulations with the help of PB, and drafted the manuscript. PB contributed in designing the study. RWP helped in interpreting the results and in drafting the manuscript. All the authors read and approved the final manuscript.
